# Positioning about the Flexibility of Fasting for Lipid Profiling

**DOI:** 10.5935/abc.20170039

**Published:** 2017-03

**Authors:** Marileia Scartezini, Carlos Eduardo dos Santos Ferreira, Maria Cristina Oliveira Izar, Marcello Bertoluci, Sergio Vencio, Gustavo Aguiar Campana, Nairo Massakazu Sumita, Luiz Fernando Barcelos, André A. Faludi, Raul D. Santos, Marcus Vinícius Bolívar Malachias, Jerolino Lopes Aquino, César Alex de Oliveira Galoro, Cleide Sabino, Maria Helane Costa Gurgel, Luiz Alberto Andreotti Turatti, Alexandre Hohl, Tania Leme da Rocha Martinez

**Affiliations:** 1 Sociedade Brasileira de Análises Clínicas (SBAC), Rio de Janeiro, RJ, - Brazil.; 2 Sociedade Brasileira de Patologia Clínica/Medicina Laboratorial (SBPC/ML), Rio de Janeiro, RJ, - Brazil.; 3 Sociedade Brasileira de Cardiologia (SBC), Rio de Janeiro, RJ, - Brazil.; 4 Sociedade Brasileira de Endocrinologia e Metabologia (SBEM), Rio de Janeiro, RJ, - Brazil.; 5 Sociedade Brasileira de Diabetes (SBD), São Paulo, SP - Brazil.

**Keywords:** Fasting / metabolism, Postprandial, Lipid profile, Reference values, Risk category

## Justifications

The review of the need of fasting for lipid profile analysis (total cholesterol, LDL-C, HDL-C, non-HDL-C, and triglycerides [TG]) is based on the following grounds:

- Since the postprandial state predominates during most of the day, the patient is more exposed to the lipid levels in this condition when compared with the fasting state. Therefore, the postprandial state may represent more effectively the potential impact of the lipid levels on an individual's cardiovascular risk.
- Measurements in the postprandial state are more practical and provide the patient a greater access to the laboratory, decreasing the number of missed working days and medical appointments due to missed tests, allowing a better assessment of the individual's cardiovascular risk.
- Blood collection in the postprandial state is safer in several circumstances and may help prevent hypoglycemia secondary to the use of insulin in patients with diabetes mellitus, or due to prolonged fasting in pregnant women, children, and elderly individuals, minimizing complications and increasing the adherence to the tests and the attendance to medical appointments.
- There are no significant differences in measurements of total cholesterol, HDL-C, non-HDL-C, and LDL-C performed in the postprandial or fasting state. Levels of TG increase in the fed state, but such increase has little relevance as far as a regular meal without fat overload is considered, with the possibility of adjustment in the reference values.^1-7^
- With a flexibility for lipid profiling, there is a greater amplitude of schedules, thereby reducing congestion in the laboratories, especially early in the morning, bringing more comfort to the patient.
- With the technological advances in diagnostic methods, the main assays available have mitigated the interference caused by increased sample turbidity due to high TG concentrations. However, there are potential limitations, especially related to the calculation of LDL-C, in which performance studies of different methodologies have shown a need for a revision of the practical use of the adopted formulas.

### Clinical and Laboratory Aspects in the Flexibility of Fasting for Lipid Profile Analysis

In easing the requirement for fasting in the collection of samples for lipid profile assessment, some clinical and laboratory recommendations become important.

### Recommendations for the care of the patient in the laboratory

- Nonfasting sample collection for lipid profiling: may be done by the laboratory with the presence of the information about fasting at the time of sample collection in the laboratory report.
- A medical request without a definition of the fasting duration and without other tests known to require fasting: it is recommended to include in the laboratory report the fasting duration informed at the time of the sample collection.
- Presence in the same request of other tests that require fasting: the laboratory may define that the lipid profile should be collected with a 12-hour fasting when other laboratory tests, ordered on the same request, also require this period of fasting. The laboratory is recommended to specify whether or not fasting is required for each exam: no fasting, 12-hour fasting, or according to the definition set by the laboratory.
- When an indication of a specific fasting duration is present: if the request by the physician has a specific fasting duration, the laboratory should follow such recommendation. The calculation of hours of fasting by the "SIL" (Laboratory Information System, *Sistema de Informação Laboratorial*) may be used, based on the information of the time elapsed since the last meal.
- When the TG levels in the postprandial state are > 440 mg/dL, or in the presence of special situations such as the recovery from pancreatitis secondary to hypertriglyceridemia, or at the beginning of a treatment with drugs that cause severe hypertriglyceridemia, the prescribing physician is recommended to request a new TG evaluation with a 12-hour fasting and this will be considered as a new TG test by the laboratory.^1^
- When the second sample collection for TG measurement occurs: the use of the same code or another specific code for the TG measurement without fasting and after a 12-hour fast will be at the discretion of each laboratory, depending on its system and strategy.

### Template recommendations for the laboratory's report

The report is a responsibility of the laboratory and its technical manager. With the purposes of alignment and harmonization among the institutions, it is recommended the adoption of the following information in the report:

- The reference values and therapeutic target for the lipid profile (adults aged > 20 years) according to the cardiovascular risk assessment estimated by the prescribing physician are described in Table 1.^1,8,9^
  **Table 1:** Reference values and therapeutic targets for adults > 20 years according to the patient’s cardiovascular risk assessed by the physician requesting the lipid profile

Lipids	With fasting (mg/dL)	Without fasting (mg/dL)	Referential category
Total cholesterol*	< 190	< 190	Desirable
HDL-C	> 40	> 40	Desirable
Triglycerides**	< 150	< 175	Desirable
			**Risk category**
LDL-C	< 130 < 100 < 70 < 50	< 130 < 100 < 70 < 50	Low Intermediary High Very high
Non-HDL-C	< 160 < 130 < 100 < 80	< 160 < 130 < 100 < 80	Low Intermediary High Very high

* Total cholesterol > 310 mg/dL: consider the likelihood of familial hypercholesterolemia;

** When the triglyceride levels are above 440 mg/dL (without fasting), the prescribing physician must request a new triglycerides measurement after 12-hour fasting and the laboratory should consider this as a new triglycerides test.
- Insertion of a note in the report referencing that the lipid profile results should be interpreted according to the clinical assessment and evolution of the patient. The following sentence is recommended: "The clinical interpretation of the results should take into account the reason for indication of the test, the metabolic state of the patient, and the stratification of risk for establishment of the therapeutic goals."
- The target reference values of the lipid profile for children and adolescents are shown in Table 2.^10,11^
  **Table 2:** Reference values of lipid profile for children and adolescents

Lipids	With fasting (mg/dL)	Without fasting (mg/dL)
Total cholesterol*	< 170	< 170
HDL-C	> 45	> 45
Triglycerides (0-9 years) **	< 75	< 85
Triglycerides (10-19 years) **	< 90	< 100
LDL-C	< 110	< 110

* Total cholesterol > 230 mg/dL: consider the likelihood of familial hypercholesterolemia;

** When the triglycerides levels are above 440 mg/dL (without fasting), the prescribing physician must request a new triglycerides measurement after 12-hour fasting and the laboratory should consider this as a new triglycerides test.
- Patients with diabetes and no risk factors or evidence of subclinical atherosclerosis should maintain LDL-C levels below 100 mg/dL. Patients with risk factors or subclinical atherosclerotic disease should maintain LDL-C levels below 70 mg/dL. Patients with a history of acute myocardial infarction; stroke; coronary, carotid or peripheral revascularization; or history of amputation should maintain the LDL-C levels below 50 mg/dL.
- The inclusion of a specific note about the screening of familial hypercholesterolemia (FH) is left at the discretion of the laboratory. The following sentence is recommended: "Values of total cholesterol > 310 mg/dL in adults or ≥ 230 mg/dL in children and adolescents may be indicative of familial hypercholesterolemia, if secondary dyslipidemias are excluded."^14^

### Recommendations about formulas and direct LDL-C measurement

The assessment of LDL-C can be performed by direct measurement or estimated by calculation based on Friedewald's or Martin's formula.^13^ The following recommendations are suggested to the laboratories:

- Observe the limitations of nonfasting and TG values > 400 mg/dL when Friedewald's formula^15^ is used to estimate LDL-C; in these cases, Martin's formula^16^ or direct measurement should be used.
- When collecting postprandial samples, the LDL-C measurement can be performed by direct measurement or calculated using Martin's formula.^16^
- Include non-HDL-C in the calculation along with other results of the lipid profile in adults, even without fasting, since the TG levels do not interfere in such calculation. Reporting or not of the VLDL-C calculation may be done at the discretion of the laboratory.

The main purpose of this document is to standardize the clinical and laboratory actions in regards to the flexibility of fasting in the lipid profile analysis across the national territory, contributing to offer security to the decision-making process by physicians and laboratories, grounded by scientific evidence.
